# Systemic Sclerosis: Elevated Levels of Leukotrienes in Saliva and Plasma Are Associated with Vascular Manifestations and Nailfold Capillaroscopic Abnormalities

**DOI:** 10.3390/ijerph182010841

**Published:** 2021-10-15

**Authors:** Angélica Mandujano, Ignacio Méndez-Ramírez, Luis Humberto Silveira-Torre

**Affiliations:** 1 Departamento de Atención a la Salud, Universidad Autónoma Metropolitana-Xochimilco, Mexico City 04960, Mexico; 2 Instituto de Investigaciones en Matemáticas Aplicadas y en Sistemas, Universidad Nacional Autónoma de México, Mexico City 04510, Mexico; imendez@servidor.unam.mx; 3 Departamento de Reumatología, Instituto Nacional de Cardiología Ignacio Chávez, Mexico City 14080, Mexico

**Keywords:** leukotrienes, cysteinyl-leukotriene, saliva, systemic sclerosis, nailfold capillaroscopy, Raynaud’s phenomenon

## Abstract

The role of leukotrienes (LTs) in the pathogenesis of systemic sclerosis (SSc) needs clarification. We analyzed the association of salivary (sa) and plasma (p) levels (pg/mL) of cysteinyl-leukotrienes (CysLT) and LTB4 with SSc vascular manifestations and nailfold capillaroscopy (NFC) in a cross-sectional study. Patients and healthy controls were evaluated for vascular manifestations and NFC. LTs were compared between groups as follows: SSc with or SSc without vascular features and controls, and by NFC parameters. Twenty SSc patients and 16 volunteers were recruited; Raynaud’s phenomenon (RP) history (SSc: saCysLT 99.4 ± 21.8 vs. controls: 23.05 ± 23.7, *p* = 0.01), RP at examination (SSc: saCysLT 129.3 ± 24.6 vs. controls: 23.05 ± 22.46, *p* = 0.01; pCysLT SSc: 87.5 ± 11.2 vs. controls: 32.37 ± 10.75, *p* = 0.002), capillary loss (saCysLT 138.6 ± 26.7 vs. 23.05 ± 21.6, *p* = 0.0007; saLTB4 3380.9 ± 426.6 vs. 1216.33 ± 346.1, *p* = 0.0005), “late” scleroderma pattern vs. controls (saCysLT 205.6 ± 32 vs. 23 ± 19.6, *p* = 0.0002; saLTB4 4564.9 ± 503.6 vs. 1216.3 ± 308.3; *p* < 0.0001) were all significant. Late patterns had higher levels (saCysLT, *p* = 0.002; LTB4 *p* = 0.0006) compared to active and early patterns (LTB4, *p* = 0.0006), and giant capillaries (*p* = 0.01) showed higher levels of LTs. Levels of pCysLT were higher in patients with RP at examination vs. patients without RP; saCysLT and LTB4 were higher in SSc group with vs. without capillary loss. LTs could be involved in the pathophysiology of vascular abnormalities. Further research is required to determine if blocking LTs could be a therapeutic target for SSc vascular manifestations.

## 1. Introduction

Systemic sclerosis (SSc) is an autoimmune disease characterized by fibrosis of the skin and some internal organs [1]. Vascular disease plays a central role in its pathogenesis. SSc vasculopathy is a progressive angiopathy with remodeling of arterial vessels and microcirculatory abnormalities, which causes local ischemia that promotes tissue fibrosis [2]. Clinical manifestations are due to organ dysfunction secondary to fibrosis, vascular damage and inflammation [2,3]. Mortality rates have proven to be high, mainly due to pulmonary fibrosis and pulmonary arterial hypertension [3,4,5]. The pathogenesis involves the interaction of numerous biochemical mediators [1,6,7]; however, compared to others, leukotrienes (LTs) have received little attention. These lipidic molecules, including LTA4, LTB4 and cysteinyl-leukotrienes (LTC4, LTD4 and LTE4), are derived from arachidonic acid through the 5-lipoxygenase (5-LO) pathway and have both inflammatory and fibrotic properties [8,9,10,11]. LTs have been implicated in several animal and human models of fibrotic disease. In humans, these include idiopathic pulmonary fibrosis (IPF), airway remodeling in asthma, bronchiolitis obliterans, asbestosis, acute lung injury, cystic fibrosis, among others [10,12]. In remodeling disorders of the lung, including SSc associated interstitial lung disease, the eicosanoid imbalance hypothesis, where profibrotic LTs predominate over anti-profibrotic prostaglandins PGE2 and PGI2 has been proposed to participate in its pathophysiology [8,13]. Among its profibrotic properties, LTs stimulate fibroblast and myofibroblast proliferation and chemotaxis, and collagen synthesis. In addition, cysteinyl-leukotrienes (CysLT) promote the proliferation of bone marrow-derived fibrocytes, which participate in tissue remodeling [8]. However, there are also growth and differentiation factors of other cell types, including lymphocytes, fibroblasts, endothelial and epithelial cells, that are actively involved in inflammation, fibrosis and vascular function [9,10,11,14,15]. LTs have been extensively studied in IPF and in their animal model, bleomicyn-induced pulmonary fibrosis mice. In this model, an important role of LTB4 and CysLT through BLT1 and CysLT_2_ receptors [13,16] in fibrosis induction has been showed, and deletion [16,17] or blocking of LTs receptors [18] or their biosynthetic enzymes, including 5-LO, LTC4 synthase [16,19], and LTA4 hydrolase [12], were related with a significant reduction of fibrosis. Evidence supports that LTs induce fibrosis by inducing transforming growth factor beta (TGF-β) release from macrophages and epithelial cells [8,17,20]. Other researchers have demonstrated in rat fibroblasts that LTB4 can induce fibroblast migrations and cell proliferation and suggested that it could also be induced by reactive oxygen species (ROS) generation [21].

Regarding the vascular activities of LTs, they have a potent vasoconstrictor effect in many vascular beds [22] including microcirculation of pulmonary vessels, rat mesenteric arterioles and coronary microvessels [23]. In some animal models, it has been reported that LTs induce vasoconstriction either directly through LTs receptors, or indirectly by stimulating other lipidic mediators such as thromboxanes and prostaglandins [23]. CysLT are potent vasoconstrictors in the lung, and their release may be important during pulmonary hypoxia vasoconstrictor response. However, there is great variability depending on the species studied [24]. It has also been shown that LTB4 is involved in microvascular response to hypoxia, through ROS generation and by favoring leukocyte-endothelial adherence [25].

In addition, CysLT are produced during vascular injury, and they modify vascular function with a wide array of effects such as constriction of microvasculature, promotion of post-capillary venules permeability, reduction of coronary flow and pathological retinal angiogenesis [26]. CysLT induce recruitment of leukocytes into the arterial wall, which contribute to vascular remodeling and thrombosis [10]. They also increase vascular permeability, induce proliferation of vascular smooth muscle and contractibility of both [27] vascular and bronchial muscle cells [10,28]. There is evidence to support that CysLT regulate endothelial function by modulating inflammation through CysLT_1_ receptor and endothelial cell proliferation through the CysLT_2_ receptor [26]. All these vascular and profibrotic mechanisms activated by CysLT suggest that they could play a key role in pathogenesis of Raynaud’s phenomenon as seen in SSc.

Cross-sectional studies have shown high levels of LTB4 and LTE4 in bronchoalveolar lavage fluid of patients with scleroderma lung disease [20,29]; high amounts of CysLT in exhaled breath condensate in SSc patients [30]; in SSc skin overexpression of 5-LO, critical in LTs synthesis; an increase in SSc fibroblast LTB4 production after stimulation with an ionophore [28]; and greater expression of 5-LO in fibroblasts, which lowers collagen production and TGF-β1 when the enzyme is inhibited [31]. In a more recent study, Liang and coworkers [32] found higher serum levels of LTB4 in SSc patients compared to controls, and these levels were associated with subsets that had interstitial lung disease and diffuse SSc. In addition, levels of leukotriene A4 hydrolase and the LTB4 receptor BLT1 were increased in areas of skin and lung sclerosis [32]. However, the role of LTs in the complex pathogenesis of SSc and their possible role as therapeutic targets remains unclear. Although LTs are involved in inflammation, vascular function and fibrosis pathways and the clinical manifestations of SSc are mainly due to abnormalities in these same processes, previous studies have not researched their possible association. SSc is a heterogenous disease [3] in terms of its manifestations and its severity, therefore we postulate that levels of LTs would be higher in patients with vascular manifestations in SSc compared to both SSc patients without vascular manifestations and healthy volunteers. The aim of this study was to quantify levels of LTs in more easily obtainable samples such as saliva and plasma and to compare such levels between the groups divided not according to the presence or absence of the disease, but by the vascular findings at clinical examination and at nailfold capillaroscopy (NFC).

## 2. Materials and Methods

Ethical Considerations. The cross-sectional study was conducted according to the guidelines of the Helsinki Declaration and local regulations, and we followed appropriate ethical research design and the relevant standards to ensure the protection of human subjects. The protocol was approved by the Research Commission and Ethics Committee of Instituto Nacional de Cardiología Ignacio Chávez (IRB: 12-776). Informed consent was obtained from all subjects involved in the study.

Patients and controls. Patients and healthy volunteers fulfilled all inclusion criteria including age greater than or equal to 18 years and agreement to participate under informed consent; neither healthy volunteers nor patients that had history of bronchial asthma, rhinitis, allergic diseases or other systemic diseases were enrolled. Patients were recruited from the Rheumatology Department at the Instituto Nacional de Cardiología Ignacio Chávez and all patients fulfilled the 2013 American College of Rheumatology/European League Against Rheumatism criteria for the classification of SSc [33], patients with limited and diffuse SSc were included. Healthy volunteers were companions (relatives or friends of the patients) or hospital staff.

**Figure 1:**
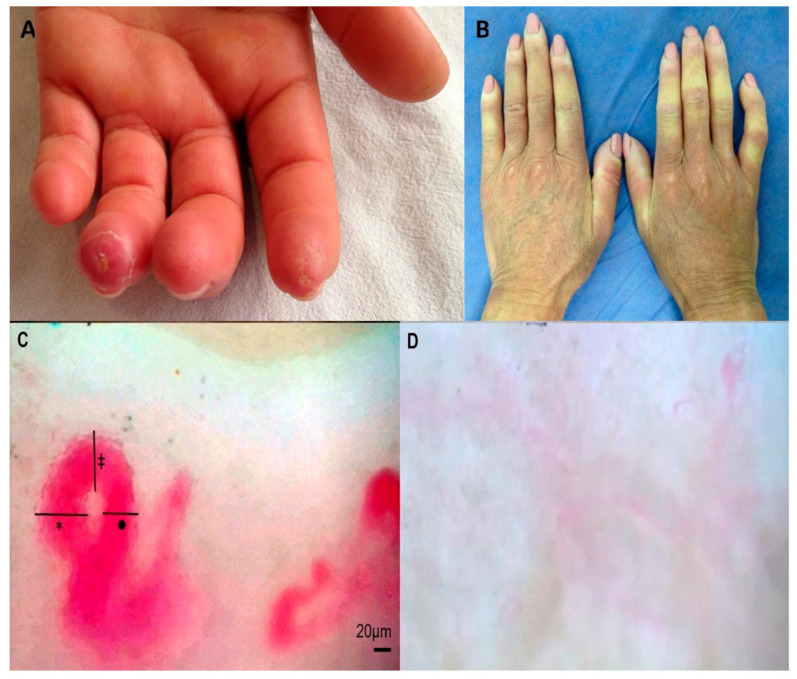
Clinical and NFC assessment. Clinical and capillaroscopic features were assessed in the study population. A representative patient with fingertip scars (**A**). A patient Raynaud’s phenomenon at examination (**B**). NFC in one of the cases included in the study, schematic representation of capillary diameters assessed in all individuals in nailfold capillaroscopy (*) arterial limb, (•) venous limb and (‡) top diameters are shown (**C**). NFC was evaluated in all individuals and classified in “early”, “active” or “late” patterns (Scleroderma patterns) or “normal” capillaroscopy; a case of “late” pattern shows severe capillary loss with wide avascular areas and severe disorganization of the normal capillary array (**D**). NFC: nailfold capillaroscopy.

Clinical assessment. A complete medical history was taken for all individuals. Demographic information and vascular manifestations found at recruitment were collected. Vascular features included Raynaud’s phenomenon (RP), digital ulcers (DU) and fingertip scars both since the onset of the disease (RP, DU and fingertip scars history) and RP, DU and fingertip scars found at the time of physical examination. Clinical photographs were also taken (Figure 1A,B).

Nailfold capillaroscopy (NFC). NFC was performed in all patients on the fourth finger of their right hand at room temperature with the patient in a sitting position. We chose to evaluate the same finger in all subjects to reduce the variability that could be found when comparing different fingers. The fourth finger was chosen for assessment because it has been previously reported that it is in this finger where the presence of enlarged capillaries and hemorrhages is most frequent in patients with SSc. Additionally, it has a greater transparency of the skin compared to other fingers [34]. This same approach has been used in other studies [35]. We used a capillaroscope model JH-1004 C with a total magnification of 380X to obtain images that were analyzed with the software MicroCirculationXP (Jiangsu Jiahua Electronic Instrument Co., Xuzhou, China). Capillary morphology, density, dimensions and architecture were registered, including apical, arterial and venous limb diameters. Capillary size was classified in all subjects as: normal capillaries (limb diameter < 20 µm), capillary ectasia (limb diameter between 20–50 µm) and giant capillaries (>50 µm). Other parameters were classified according to previously published parameters (Figure 1C,D) [35].

Primary endpoint. The primary endpoint was to detect any differences in LT levels between the groups classified by vascular features and capillaroscopy findings. Other endpoints. Levels of LTs between patients and healthy volunteers were compared, and LT levels by clinical groups were also analyzed. In addition, we looked for correlation between capillaroscopy parameters and LT levels.

Procedures. All samples (saliva and plasma) were collected between 7:00 and 9:00 after fasting on the same day of clinical assessment. Peripheral venous blood was collected in sodium heparin BD Vacutainers. Non stimulated saliva was collected from most subjects between 7:00 and 9:00 on the same day before clinical evaluation. Only patients with insufficient saliva chewed paraffin gum to take a stimulated saliva sample. Patients and controls were asked to continuously spit out all their saliva for 5 min in plastic cones connected to 15 mL polypropylene centrifuge tubes (corning) according to a previously published method [36].

Enzyme immunoassay (EIA). Peripheral blood and saliva samples were collected, centrifuged at 2600× *g* for 15 min at 4 °C and stored at −80 °C until processing. The LTs in all samples were identified and quantified by analytical services of Cayman Chemical in Ann Harbor, Michigan, USA. Levels of LTB4 and CysLT in plasma and saliva were determined with Cysteinyl Leukotriene Express EIA and Leukotriene B4 Express EIA kits in triplicate. The Cayman kit protocols were followed by the analytical services. For CysLT and LTB4 assays for saliva and plasma, samples were purified using the Purification Protocol for each type of kit to avoid contaminants, which can interfere in the assay.

Statistical analysis. Demographic, clinical, and laboratory characteristics of SSc patients were summarized as mean ± standard error of the mean or number (%). Associations between participant groups and LTs and clinical findings were analyzed by ANCOVA, with patient age as a covariate. The following procedure was used to create the models. First, an ANCOVA model in which the participant groups were the explanatory variable, the subject’s age the covariate, and the product of these two as the interaction term was constructed for each of SSc-related variables as the respective outcomes; second, ANCOVA model assumption sufficiency was tested through the goodness of fit of studentized residuals to normal distribution. When this assumption was satisfied, the ANCOVA model was subjected to the variable selection procedure by the backward stepwise method. In the case it was not, the Box-Cox transformation was applied to the outcome variable. After these steps, if the effect of the explanatory variable, subject groups, was statistically significant, the differences among groups were determined by Tukey’s honestly significant difference test (Tukey’s HSD). For all statistical tests, *p* < 0.05 was recognized as statistically significant. We used the Holm–Bonferroni Sequential Correction procedure to protect against Type I errors for correlation analyses. Correlations were evaluated by the Spearman rank test. Data analysis was performed using JMP software, version 13.0 (SAS Institute, Cary, NC, USA) and an excel calculator version 1.2 (2013) to calculate the Holm–Bonferroni sequential correction [37].

## 3. Results

Twenty patients (12 with limited and 8 with diffuse SSc) and 16 healthy volunteers were recruited, and the baseline characteristics of subjects, clinical features and vascular manifestations are shown in Table 1. Only female patients were included in this study because of the expected female preponderance for disease. Only one male patient with SSc was located during recruitment, but did not agree to participate in the study. Two of the saliva samples of SSc patients were not large enough to measure CysLT and LTB4 concentrations, despite patients being asked to chew paraffin gum. Only 3/20 patients and 1/16 of healthy volunteers required a new stimulated saliva collection to obtain appropriate sample.

**Figure 2:**
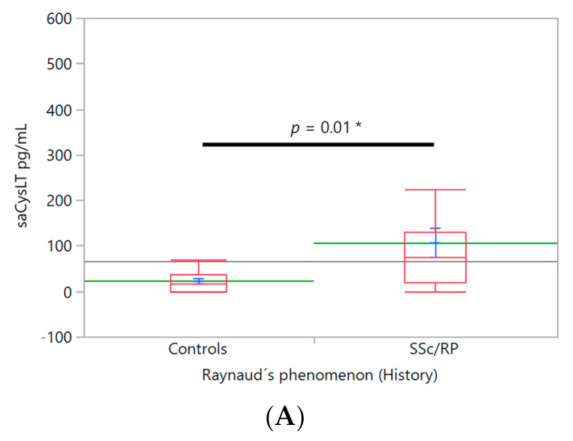

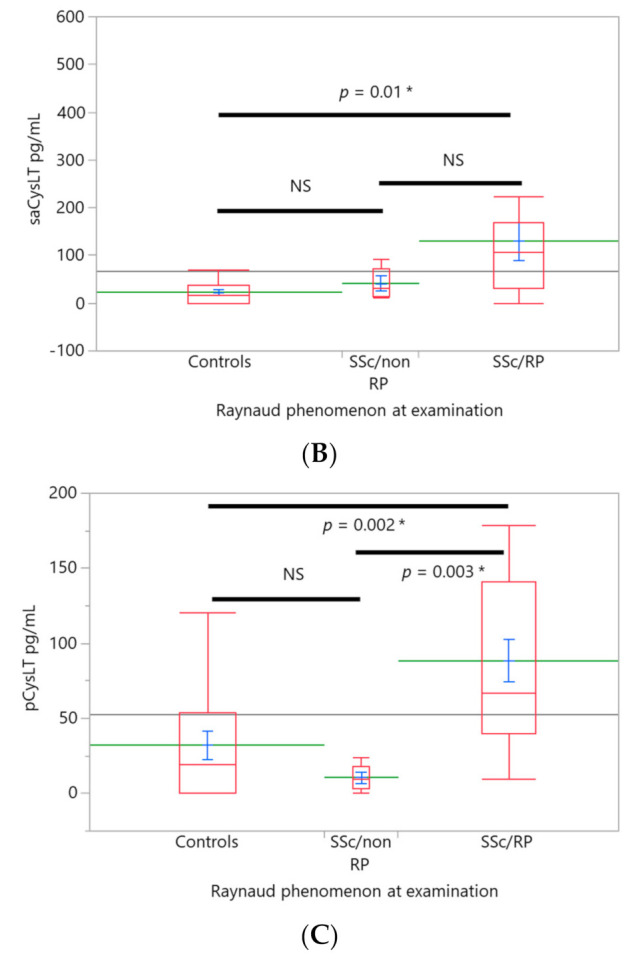
LTs levels and vascular manifestations. Differences in levels of leukotrienes according to the presence or absence of vascular manifestations that showed statistical differences. Box plots (red), mean (green), mean error bars (blue) and grand mean (grey line across the boxes) are shown. The X axis is proportional. Overlap marks are drawn to illustrate statistical differences between the groups. Levels of CysLT in salivary samples in SSc with RP history were compared to controls (**A**). saCysLT levels between the groups of SSc with (SSc/RP) and without (SSc/non RP) RP at examination and control group were compared (**B**). Levels of pCysLT were compared between groups: with or without RP at examination, and control group (**C**). Analysis of covariance (ANCOVA) was used for analysis and Tukey HSD test was used to detect the differences between all pairs. RP: Raynaud´s phenomenon, LTs: leukotrienes, CysLT: cysteinyl-leukotrienes, sa: salivary, p: plasmatic, NS: non-significant, *: to denote statistically significant test.

LTs and vascular abnormalities. Patients with RP history showed higher levels of salivary CysLT (99.4 ± 21.8 pg/mL) compared to controls (23.05 ± 23.7 pg/mL, *n* = 16), F(1,32) = 6.33, (Figure 2A). Only one patient had no history of RP, so it was eliminated from the analysis. Two salivary samples were insufficient to measure salivary CysLT and LTB4 (*n* = 17). SSc patients with RP at the time of examination had higher levels of salivary CysLT (129.3 ± 24.6 pg/mL, *n* = 13) compared to controls (23.05 ± 22.46, *n* = 16), but not compared to patients without RP at examination (41.35 ± 40.2 pg/mL, *n* = 5), F(2,33) = 5.31 (Figure 2B). A similar trend was found in plasmatic CysLT, patients with RP at examination (87.5 ± 11.2 pg/mL, *n* = 15), compared to SSc without RP at examination (10.73 ± 19.24 pg/mL, *n* = 5 Figure 2C) and controls (32.37 ± 10.75, *n* = 16), F(2,33) = 5.31. With plasma, all samples were analyzed.

**Figure 3:**
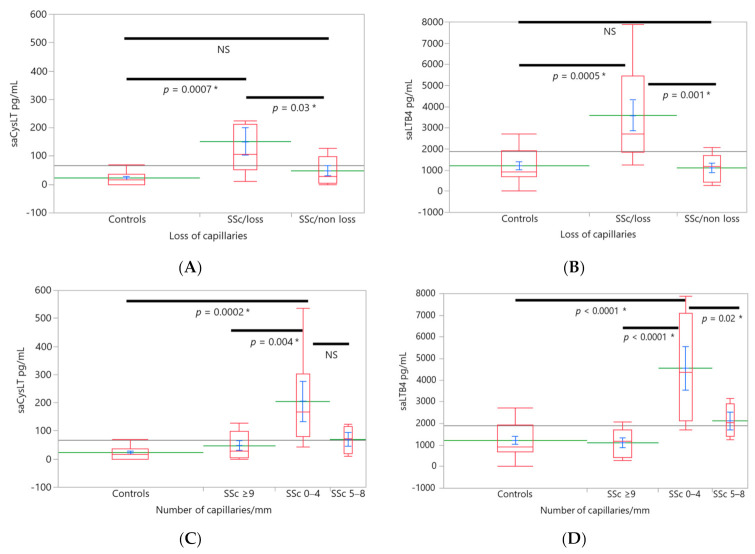
LT levels and NFC findings. Levels of leukotrienes were associated with abnormal nailfold capillaroscopy. Box plots (red), mean (green), mean error bars (blue) and grand mean (grey line across the boxes) are shown. Overlap marks are drawn to illustrate statistical differences between the groups. The X axis is proportional. Levels of salivary CysLT (**A**) and LTB4 (**B**) were higher in the SSc group with capillary loss compared to SSc group without loss and controls (capillary number ≥ 9 capillaries per millimeter). Levels of salivary CysLT (**C**) and LTB4 (**D**) were higher in the group with a number of capillaries between 0–4 per millimeter compared to the group with SSc with a normal number of capillaries (≥9 capillaries/millimeter) and control group. In addition, salivary LTB4 were higher compared to SSc group with 5–8 capillaries/mm. Analysis of covariance (ANCOVA) was used for analysis and Tukey HSD test was used to detect the differences between all pairs. SSc: systemic sclerosis, LTs: leukotrienes, saCysLT: salivary cysteinyl-leukotrienes, saLTB4: salivary leukotriene B4, NFC: nailfold capillaroscopy, NS: non-significant, *: to denote statistical differences.

LTs and NFC abnormalities. Loss of capillaries. Salivary CysLT (138.6 ± 26.7 pg/mL, *n* = 11) levels were significantly higher in the SSc group with capillary loss compared to SSc without capillary loss (37.96 ± 30.5 pg/mL, *n* = 7) and controls (23.05 ± 21.6 pg/mL, *n* = 16), F(2,33) = 7.0 (Figure 3A). A similar trend was found in LTB4, SSc with capillary loss (3380.9 ± 426.6 pg/mL) was significantly higher than LTB4 levels of SSc without loss (1097.12 ± 489.45) and controls (1216.33 ± 346.1 pg/mL), F(2,33) = 10.7 (Figure 3B).

**Figure 4:**
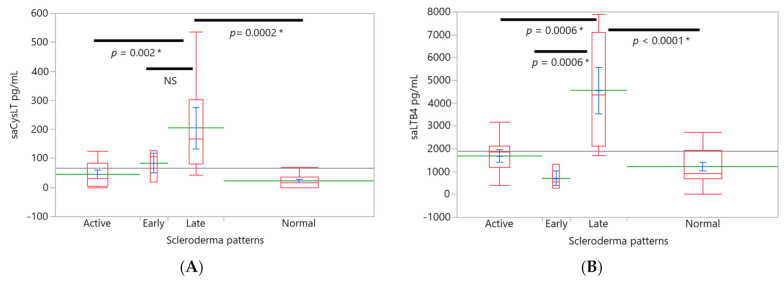
LT levels and capillaroscopy scleroderma patterns. Levels of leukotrienes were associated with scleroderma patterns. Box plots (red), mean (green), mean error bars (blue) and grand mean (grey line across the boxes) are shown. Overlap marks are drawn to illustrate statistical differences between the groups. The X axis is proportional. Levels of salivary CysLTs (**A**) and LTB4 (**B**) were higher in the group with “late” pattern compared to normal and active nailfold capillaroscopy. In addition, levels of salivary LTB4 in “late” pattern were higher than in the “early” pattern. Analysis of covariance (ANCOVA) was used for analysis and Tukey HSD test was used to detect the differences between all pairs. saCysLT: salivary cysteinyl-leukotrienes, saLTB4: salivary leukotriene B4. *: to denote statistical differences.

Amongst the groups stratified by the number of capillaries, the group with the highest capillary loss (0–4 capillaries/mm, *n* = 7) had the highest salivary CysLT (177.8 ± 32 pg/mL) levels compared with groups with SSc without loss (47.96 ± 27.93 pg/mL) and controls which had salivary CysLT of 23.05 ± 19.75 pg/mL, F(3,33) = 7.97 (Figure 3C). A similar trend was found for salivary LTB4. The highest levels were found in the group with 0–4 capillaries/mm (4103.4 ± 498.9 pg/mL) compared to SSc patients with a lesser loss (LTB4 2116.45 ± 612.54 pg/mL) and SSc patients without capillary loss (1097.12 ± 433.13) and controls (1216.33 ± 306.27), F(3,33) = 12.3 (Figure 3D). Capillary size. Three patients showed a severe loss of capillaries thus, the capillary diameter could not be assessed in them. The group with giant capillaries (*n* = 9) had the highest levels of salivary LTB4 compared to ectasia SSc group and controls, F(3,30) = 8.98; giant capillaries vs. ectasia capillaries (*p* = 0.001) and giant vs. controls (*p* = 0.01). However, in this case the ANCOVA model was also significant for age (*p* = 0.03). SSc patients with dilated capillaries showed higher levels of salivary LTB4 were compared to controls (*p* = 0.04). *Scleroderma patterns*. Patients with the “late” pattern (*n* = 6) had higher salivary CysLT (205.6 ± 32 pg/mL, Figure 4A) F(3,33) = 8.18 and salivary LTB4 (4564.9 ± 503.6 pg/mL, Figure 4B) levels compared to normal (*n* = 16) capillaroscopy (salivary CysLT 23 ± 19.6 pg/mL, LTB4 1216.3 ± 308.3 pg/mL) and compared to “active” pattern (salivary CysLT 45.49 ± 26.18 pg/mL, LTB4 1681 ± 411.18, *n* = 9). In addition, LTB4 levels were higher in patients with the “late” pattern than in patients with an “early” pattern (*n* = 3, salivary LTB4 704.4 ± 712.1 pg/mL, Figure 4B) F(3,33) = 8.18. There were no differences between the groups by age nor body mass index.

Levels of LT between the SSc and control groups. Higher CysLT concentrations in saliva and plasma were found in SSc patients (data not shown), but no statistical significance was found.

Leukotrienes and clinical groups. Patients were classified according to skin involvement in diffuse (dcSSc) and limited (lcSSc) subsets, and the level of leukotrienes was compared between the subsets and controls, but no significant differences were found.

Correlations between leukotrienes and demographic variables. Correlations between age and body mass index was not found (data no shown). Correlations between LTs. Salivary levels of LTB4 and CysLT were positively correlated (ρ of Spearman 0.51, *p* = 0.001), no other correlations between LTs were found. Correlations between leukotrienes and NFC. Levels of salivary CysLT were negatively correlated with the number of capillaries (ρ of Spearman −0.61, *p* ≤ 0.0001).

## 4. Discussion

Considering the relationship between LTs and vascular function, we found significantly higher concentrations of LTs in SSc patients that had vascular features. We found higher levels of salivary and plasma CysLT in patients with RP and higher salivary levels of both LTB4 and CysLT were associated with loss of capillaries and “late” scleroderma pattern. In addition, higher levels of salivary LTB4 were associated with giant capillaries and dilated capillaries. Such features are caused by tissue hypoxia, one of the pathogenic mechanisms of SSc. This suggests that they could be related with LTs synthesis as these findings are consistent with the reported biological activity of leukotrienes [9,10,14,15,38,39].

To our knowledge, few studies have focused on a potential association between RP and LTs. Lau and colleagues [40] investigated the possible relationship between inflammatory properties of LTB4 and RP in patients with SSc and vibration induced white syndrome and found an increase of both ROS generation and LTB4 by stimulated polymorphonuclear cells in both groups compared to normal controls. In another study Azevedo et al. [41] assessed the effect of LTs receptor antagonist montelukast on vascular alterations in NFC of patients with RP. They found that anti-leukotriene therapy was associated with a reduction of edema and pallor and with normalization of capillary number, size and distribution. There are no other studies on the relationship between vascular abnormalities and LTs in SSc, but several studies have addressed the relation of LTs to some hypoxic diseases. Such studies have found increased production of LTs in relation to nocturnal oxygen desaturation severity and intermittent hypoxia in obstructive sleep apnea syndrome [42,43]. Results from some animal models show that LTs could potentiate the acute and sustained phases of the hypoxic pulmonary vasoconstriction response and may be related with development of pulmonary hypertension associated with chronic hypoxia [44]. In addition, it is postulated that intermittent hypoxia stimulates LTs synthesis and pro-inflammatory molecules derived from the 5-LO pathway and then lead to atherosclerosis, cardiac ischemia and reperfusion injury [45,46,47,48,49]. Some clinical studies have found higher urinary levels of LTE4 in patients with acute myocardial ischemia [50] and higher levels of plasmatic LTB4 in patients with coronary artery disease compared with healthy controls [51]. Nobili and colleagues [52] suggested that the vasoconstrictor and pro-inflammatory effects of chronic and acute CysLT signaling could aggravate myocardial hypoxia and inflammation during bouts of hypoxia in chronic ischemic heart disease. In addition, it has been showed in endothelial cell cultures under hypoxic conditions, an enhanced expression of 5-lipoxygenase activating protein (FLAP), because of the activation of NADPH-oxidase, phosphatidylinositol-3 kinase (PI3K), mitogen-activated protein kinase (MAPK), Nuclear factor-κB and hypoxia-inducible factor (HIF)-1α suggesting possible mechanisms that lead to the generation of LTs in hypoxic diseases [53]. Interestingly, hypoxia leads to an increase of ROS and HIF1-α both required to produce TGF-β, which is induced by hypoxia [54]. Although it has been reported that skin specimens of SSc patients do not show an increase of HIF-α response [55], the relationship between these two mediators and ROS generation with LTs seems a promising line of research. Hypoxia is related to the induction of extracellular matrix proteins but the exposure of dermal fibroblast to hypoxia for less than 6 h is not associated with stabilization of HIF-1α nor induction of extracellular matrix proteins. This has been suggested to explain why primary RP is not related to fibrosis, which differs from SSc where chronic hypoxia is associated with loss of capillaries and progressive fibrosis [56]. Based on this prior knowledge, we postulate that similar mechanisms could occur as a result of chronic hypoxia in RP, which eventually leads to capillary loss and abnormalities of the nailfold capillary anatomy given that the findings observed in this study are consistent with the biological activity that has been mentioned of LTs.

Previous studies suggest a link between LTs and atherosclerosis [46,47,48,57,58,59]; LTs in the vascular wall determine early lipid retention, foam cell accumulation and intimal hyperplasia [59,60]. Moreover, LTC4 and LTD4 are potent stimuli for the release of von Willebrand factor [46,61] and other prothrombotic and vasoactive factors such as thromboxane [62], platelet-activating factor [46], P-selectine expression [47] and could induce adherence of human neutrophils [39,63], which may be relevant to hemostasis, thrombosis and vascular injury [39]. Leukotriene receptor activation on vascular smooth muscle cells is associated directly and indirectly with vasoconstriction and intimal hyperplasia [11,27,47,63,64]. All these mechanisms could contribute to the pathogenesis of RP.

Previous research supports LTs synthesis by cells of the vascular system [57,65,66] and the expression of LTs receptors in human endothelial cells [15,46,67]. Moreover, conjugation of LTA4 with glutathione to form LTC4 can take place in structural cells of the vascular wall and in platelets [38,61] and can cause leucocyte adhesion to endothelial surface [11,15]. Vascular alterations caused by SSc include RP, pulmonary arterial hypertension, atherosclerosis, coronary arterial disease, intimal hyperplasia and prothrombotic responses, so LTs could participate in association with other mediators in generating these abnormalities.

The use of saliva to test levels of LTs has several advantages, the main one being the lack of harm to the patient [68]. The high concentrations of LTB4 in human saliva contrast with its nearly undetectable levels in blood, and its role in human saliva has been difficult to explain. However, previous observations suggest that salivary LTB4 may not only have local actions in the oral cavity, but may also impact and regulate the function of tissues in the gastrointestinal tract [69]. Whether the LTs are secreted by the salivary glands, exchanged over the ductal epithelium or synthesized by cells of the oral cavity remains unclear [70]. We also need to elucidate whether the vascular features associated with higher levels of LTs are the result of a local or a systemic process, but we postulate that associations of plasma and mainly salivary LTs levels with clinical vascular features and capillaroscopic abnormalities point to a systemic process.

Whether the LTs found in saliva and plasma participate in cell recruitment or result from the production by cells recruited by other cytokines is still an open question, but current knowledge from multiple research groups suggests that LTs could somehow be involved in the complex network of mediators participating in SSc pathogenesis [14,20,27,28,29,30,31,41].

As far as we know, only Liang et al. [32] have researched the effect of blocking the LTB4 pathway. They examined the fibroblast-myofibroblast and endothelial-mesenchymal transitions in cell cultures and found that the serum from SSc patient’s treatment, up-regulated levels of type I collagen, α-smooth muscle actin (α-SMA) and related genes, whereas in healthy control serum no changes were found. However, such changes were reversed when culture cells were treated with a selective BLT1 antagonist. Based on their previous work, they identified that the PI3K/Akt/mechanistic target of rapamycin (mTOR) signaling was implicated in myofibroblast differentiation in SSc patients and Akt is also implicated in BLT1 signaling. They then evaluated the pretreatment human dermal fibroblasts and human umbilical vein endothelial cells (HUVEC) with the mTOR inhibitor rapamycin or with a PI3K/Akt inhibitor and found that up-regulation of a α-SMA and type I collagen induced by LTB4 was reversed. The effects of exogenous LTB4 on downregulation of expression of endothelial in HUVEC (E-cadherin and CD31) and up-regulation of α-SMA, fibronectin, collagen 1A2 and 3A1 genes in both culture cells were also reversed. In addition, they confirm those results using siRNAs to knockdown Akt and mTOR. Exogenous LTB4 induced phosphorylation of Akt and mTOR and was found to be BLT1-dependent because knockdown or pretreatment with an BLT1 inhibitor blocked Akt and mTOR phosphorylation. To ensure results were not due to TGF-β, its levels were measures. They found it to be under limit of detection in culture medium. Additionally, the treatment with a selective inhibitor of TGF-β receptor I failed to affect the expression of the genes mentioned above. As a result of those experiments, they suggest that inhibition of myofibroblast differentiation could be mediated by interfering with the LTB4-BTL1 axis and the inactivation of PI3K/Akt/ mTOR signaling independently from TGF-β [32]. In addition, in mice with bleomycin-induced SSc model, the treatment with a BLT1 antagonist in an early stage show some antifibrotic effects in the skin and lungs. Similar results were observed in a late stage, but the collagen content in the skin did not reach statistical differences compared to the control group [32]. We hypothesize that blocking LTs may provide a novel strategy to treat vascular manifestations of SSc, considering that LTs exert direct effects on the vascular system but also on the late stages of disease. Vascular manifestations are caused not only by imbalance on the vascular tone between vasodilatory and vasoconstrictive mediators, but also with structural changes in the microcirculation [1,71], where LTs could be participating. In this regard, previous studies suggest that the endothelial-to-mesenchymal transition process by which endothelial cells express mesenchymal cell products such as collagen type I and α-SMA favors a profibrotic phenotype in the pathophysiology of SSc [72]. In fact, the BLT1 receptor is abundant in endothelial cells and LTB4 directly promotes myofibroblast differentiation from endothelial cells via BLT1 and PI3/Akt/mTOR signaling [32]. However, this disease is most likely to require a therapeutic approach involving the blockade of multiple targets and stimulation of others. It is not surprising to expect a complex treatment in SSc, considering it’s a complex disease; however, one of the targets could include LTs. The treatment of vascular manifestations becomes relevant not only because it occurs in almost all SSc patients, but also because in many cases it is very difficult to treat. In addition, RP is associated with morbidity and severe complications such as gangrene, reduced health-related quality of life and disability. Other limitations include pharmacological adverse events (for example of vasodilatory drugs) [71] and some of the treatments being too expensive and not widely available for low-income patients. Therefore, antileukotriene therapy in vascular manifestations of SSc is an open field to study, but further studies are required to analyze the potential of therapeutic targets.

Although a modest size and cross-sectional design of the study are potential limitations, a follow-up study is planned to assess whether the findings hold over time and are associated with disease progression. We are aware of other limitations of the present study. An important limitation is that some patients were under immunosuppressive treatment, so we included the treatment as a covariable and did not find differences between levels of LTs in the groups analyzed. In addition, it is important to mention that many of the patients evolve despite of current SSc treatments. Therefore, we postulate that even though some patients have immunosuppressants, which can affect in some degree the levels of inflammatory mediators, if LTs have a role in pathogenesis in SSc, on the one hand their levels would be unaffected or could be only partially affected since they are not specific antagonist blockers of LTs and on the other hand it may have a complex interaction with other mediators of the disease, so this could explain why the disease in many cases evolves despite of the current immunosuppressive treatment.

The value of the present study lies in two main issues. Firstly, it provides the observational data for a generation of new hypotheses regarding the pathophysiology of the vascular and cutaneous manifestations of this autoimmune disease, with possible implications in its treatment. Secondly, we believe the present study shows the importance of stratifying patients according to their clinical profile in order to identify differences in the study variables. In this study, we found similar levels of LTs between asymptomatic patients and controls, the same was observed for NFC parameters. Those who had normal or few abnormalities had similar levels to the controls. However, symptomatic patients or with important abnormalities in NFC showed higher levels of LTs pointing to a possible role in their pathogenesis.

## 5. Conclusions

CysLT and LTB4 levels, mainly in saliva samples, were associated with RP and capillaroscopic manifestations of SSc. Saliva proved to be a useful source of LTs to study their involvement in SSc.

The results are further confirmation of the potential involvement of LTs as mediators in SSc and encourage translational research on the role of LTs to find out their relevance in this complex pathogenesis, focusing on the processes of vascular damage and fibrosis in SSc.

Including LTs in the context of SSc is very important, not only from the point of view of pathogenesis, but also to consider possible combined treatment strategies to improve survival and life quality for patients.

## Figures and Tables

**Table 1:** Baseline characteristic of SSc group at recruitment.

Variable	SSc	Controls
Age, mean (range), years	48.9 (18–61)	36.8 (20–69)
BMI Kg/m^2^, mean	24.4	23.6
Sex, females/males, *n*	20/0	16/0
Smoking history, *n*	1	1
Disease duration since diagnosis mean (range), years	6.64 (0–27)	-
TSS (range), mean	14.4 (2–41)	0
dcSSc/lcSSc, *n*	8, 12	0
RP history, *n*	19	0
RP at examination, *n*	15	0
DU history, *n*	6	0
Fingertip scars, *n*	13	0
SLD, *n*	3	-
PAH, *n*	8	-
ANA, *n*	20	-
Scl-70, *n*	4	-
ACA, *n*	11	-
Treatment		-
Micofenolic acid, *n* (dose range)	8 (0.5–2 g)	
Methotrexate, *n* (dose range)	6 (12.5–15 mg)	
Prednisone, *n* (dose range)	2 (10–15 mg)	
Nifedipine, *n*	7	
Angiotensin receptor blockers, *n*	3	
Angiotensin-converting enzyme inhibitors, *n*	3	
Acetylsalicylic acid, *n*	2 (100 mg)	
Capillaroscopy, scleroderma patterns, *n*		
Early	5	0
Active	10	0
Late	5	0

The control group included healthy volunteers, the subjects did not take any treatments and no serological tests were performed. SSc: systemic sclerosis, dcSSc: diffuse cutaneous systemic sclerosis, lcSSc: limited cutaneous systemic sclerosis, BMI: body mass index, modified Rodnan TSS: total skin score, SLD: scleroderma lung disease, ANA: antinuclear antibodies, Scl-70: anti-topoisomerase I antibodies, ACA: anticentromere antibodies.

## Data Availability

Not applicable.
